# Early and progressive mobility in a community hospital: A new interdisciplinary safe patient handling and mobility model

**DOI:** 10.1097/01.NUMA.0000919068.76409.b2

**Published:** 2023-02-28

**Authors:** Danielle Gabele, Sheriee Mendez, Karen K. Giuliano

**Affiliations:** **Danielle Gabele** is the chief nurse executive at Ventura County Medical Center and Santa Paula Hospital in Ventura, Calif., and a former CNO at Cedars Sinai Marina del Rey Hospital in Marina del Rey, Calif. **Sheriee Mendez** is the program manager, Safe Patient Handling and Falls at Cedars Sinai Marina del Rey Hospital in Marina del Rey, Calif. **Karen K. Giuliano** is a professor and the co-director of the Elaine Marieb Center for Nursing and Engineering Innovation at the University of Massachusetts Amherst in Amherst, Mass.

## Abstract

An early mobility program in a small community hospital resulted in high levels of staff engagement and decreased rates for falls and heel and sacral pressure injuries.

**Figure:**
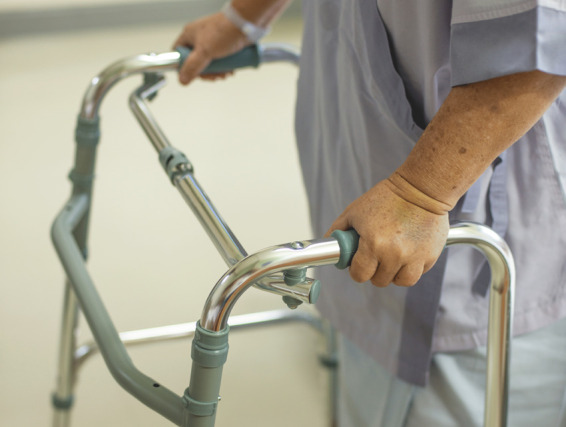
No caption available.

Early mobility (EM) programs have been recognized for improving nurse-sensitive patient outcomes, but implementation varies across organizations. One community hospital implemented a formal EM program that incorporated the use of trained mobility technicians, an established safe patient handling program, and a novel enhanced Bedside Mobility Assessment Tool (BMAT) to understand its impact on patient outcomes and staff workflow.^1^,^2^ Postintervention reductions in patient falls, heel and sacral pressure injuries, and patient-handling–related caregiver injuries suggest that this new model is a strong example of a successful EM program in a small institution.

## Background

EM is defined as patient activity, including both passive and active movement, that's initiated shortly after patient admission or mechanical ventilation. Literature promoting EM as a safe and feasible intervention grew in the early 2000s and recent studies continue to demonstrate the many positive effects of mobilizing patients early and often.^3^,^4^ Data support numerous benefits of formal EM programs, including reductions in patient length of stay (LOS), delirium, pressure injuries, and functional mobility decline during acute care hospitalization.^5^-^7^

Despite the well-known benefits of EM, a review of the literature didn't find any consistently effective strategies for implementation of EM across the various studies.^3^-^8^ As a result, the published benefits of mobility vary greatly. Programs led by nurses, therapists, or with a team approach all demonstrate different outcomes for patients.^4^ Recent articles advocate for the use of a trained mobility technician, who provides ongoing safe patient handling and mobility (SPHM) staff training and mobility assistance at the point of care.^8^,^9^ Data support that the use of mobility experts at the point of care can improve patient and staff safety and reduce hospital costs associated with caregiver injury.^9^ Additional staff support is especially critical when nurses are underresourced, such as during the recent COVID-19 pandemic.

In addition to providing physical mobility support, trained mobility technicians provide continuous education to frontline staff on the use of safe patient handling equipment, including slings, ceiling lifts, and sit-to-stand devices, which are designed to minimize the physical effort required to move patients.^10^ EM programs that use safe patient handling equipment correctly can decrease staff injury and improve staff engagement.^9^ The correct use of safe patient handling equipment can be further aided by formal assessment tools, such as the BMAT. This validated nurse-led tool is used to routinely assess patient mobility levels and guide nurses to select appropriate mobility interventions and equipment.^6^ However, no studies to date have reported the applicability of the BMAT in the community hospital setting.

Research on EM programs often focuses on the large academic hospital environment. In larger settings, resources may include lift teams, more readily available safe patient handling equipment, and robust EM protocols. Staff in smaller settings often face the challenge of limited shared resources, and patients in these facilities are at risk for immobility.^6^ Therefore, solutions are needed to strengthen patient EM across *all* institutions. The purpose of this article is to explore the impact of an EM program in one small, community hospital on patient outcomes and staff engagement and workflow.

## Methods

### Setting

This quality improvement program was implemented by the CNO on the ICU and medical-surgical floors of a 133-bed urban community hospital (with an average daily census of 60 patients) in the western US. The desire to implement a new EM program arose, in part, from the need to support nursing staff during the COVID-19 pandemic. Increased burdens on nursing staff and increasing pressure on limited resources has reduced the use of EM, with too many patients spending much of their in-hospital time in bed when not working with physical therapy. The safe patient handling devices often sat unused in various corners of the hospital. Like many hospitals at the height of the pandemic, patient outcomes began to decline. The community hospital was struggling with a high incidence of falls and hospital-acquired pressure injuries.

### EM program implementation

The CNO first enlisted the help of an external vendor who provided mobility technician services to the nursing staff between February 2020 and February 2022. During the 2-year period with the vendor service, the hospital provided training to all frontline nursing staff throughout the hospital, including RNs and ancillary nursing staff. This training focused on ergonomics and appropriate safe patient handling equipment use; it prepared nurses for a new EM program by creating standards and routines that included mobility as part of the standard of care.

**Figure 1:**
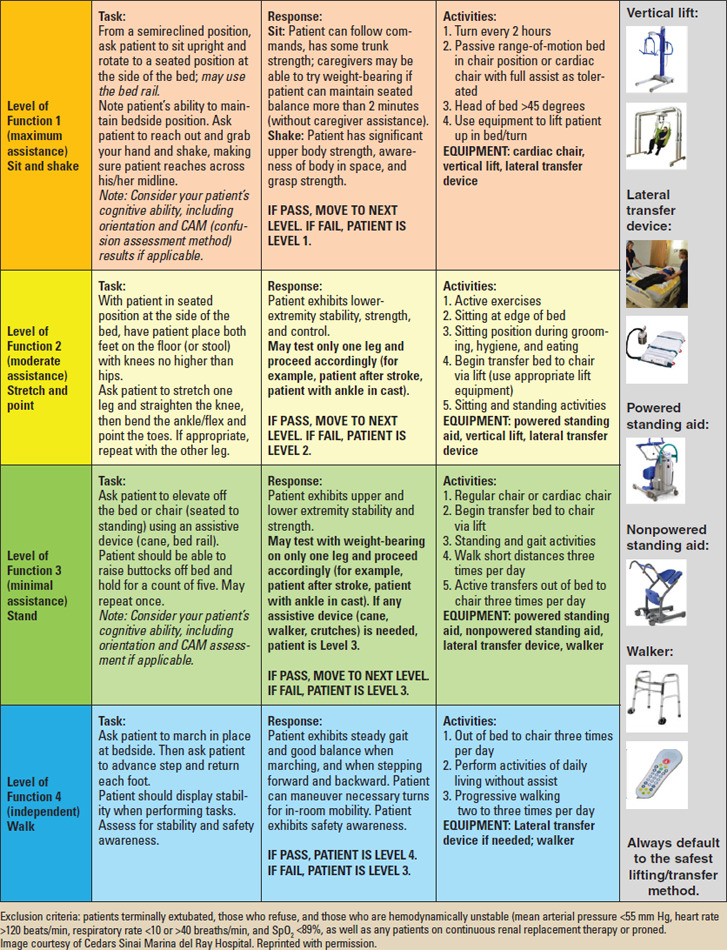
Mobility assessment tool (adapted from the BMAT)^1^,^2^

Implementation of the formal EM program was accomplished by mid-2021. In addition to the presence of EM technicians and the hospital's preexisting safe patient handling program, a novel crosswalk tool was developed and introduced in the ICU. A crosswalk image was designed to accompany the BMAT document to illustrate appropriate interventions and equipment for each mobility level (see Figure 1).^1^,^2^ This tool aimed to assist nurses in implementing safe EM for all patients according to their individual capabilities.

Every morning, the mobility technician would arrive in the ICU and round on each patient with the primary nurse, assisting with the appropriate activity based on the nurse assessment of BMAT level. Two mobility technicians were available for 8 hours per day, Monday through Friday, to support the program. In the evening, the charge nurse would repeat the same process with the frontline nursing staff, ensuring patients had mobility assessed and activity performed at least once per shift. Once the mobility technician had completed rounds in the ICU, they rounded throughout the medical-surgical areas and assisted the nurses and nursing ancillary staff who requested help. The interdisciplinary team was also supported by physical therapists, who specifically focused on mobilizing the facility's postsurgical patients.

It's important to clarify that the mobility technicians aren't a lift team. Although they do help provide mobility, they function as safe patient handling experts with a focus primarily on education for frontline staff. The mobility technicians get their patient assessment information from the nurse, complete the BMAT, independently evaluate the patient, and help select the appropriate safe patient handling supplies and equipment to best support each patient's mobility goals. The mobility technicians will demonstrate proper use of the equipment, and then work with (not for) the nurse to move the patient.

To keep the staff engaged, the CNO and program manager partnered with the mobility technician to provide multiple nursing unit trainings on the use of the lift equipment and the BMAT. The program manager routinely sent recognition emails to staff members who were strong advocates for patient mobility. Finally, an SPHM committee was formed and met monthly to discuss successes and opportunities and to track equipment use. Frontline staff was encouraged to participate in these monthly meetings.

### Program analysis

This project applied a retrospective review of patient and staff outcomes in the year prior to the EM program implementation and compared findings with outcomes once program implementation was complete. The 12-month data collection periods were based on the facility's fiscal calendar, starting July 1 and ending the subsequent June 30. Both fiscal year (FY) 2021 and FY 2022 were reviewed. Primary outcomes for both patients and staff included: 1) fall rates, 2) incidence of sacral and heel pressure injury, 3) caregiver injuries from patient handling, and 4) staff engagement scores.

1. Falls data were obtained from the hospital's incident reporting system and converted to the indicator rate of falls per 1,000 occupied bed days.
2. Hospital-acquired sacral and heel pressure injuries were tracked and reported by the hospital's wound and ostomy nurse. These injuries included all stages of pressure-related wounds identified by the wound and ostomy nurse that weren't present on patient admission.
3. Caregiver injuries related to safe patient handling were captured in the hospital's incident management system but underwent further review by the mobility technician to validate that unsafe lifting contributed to the injury. The only available data on safe patient handling injuries prior to the program onset were in FY 2019 (July 1, 2018 to June 30, 2019).
4. The facility completed two employee engagement surveys during this period that included an overall engagement index score. The facility began the engagement survey process in April 2021 and results were compared to February 2022.

## Results

### 1. Falls

In FY 2020, the fall rate was 1.849 per 1,000 occupied bed days. In FY 2021, the fall rate climbed to 2.459. In FY 2022, the fall rate declined by 53% to 1.402, which is the lowest rate achieved in the last 10 years at the hospital.

### 2. Hospital-acquired sacral and heel pressure injuries

In FY 2021, 12 hospital-acquired wounds to the heel and sacrum were reported. In FY 2022, the incidence dropped to 9 wounds.

### 3. Staff injuries

In FY 2019, there were a total of 15 patient-handling–related injuries. During FY 2021, when the mobility technician model was in place, there were a total of six patient-handling–related injuries. In FY22 only three patient-handling injuries were reported.

### 4. Employee engagement scores

In April 2021, 82% of the nursing staff reported being highly engaged. In February 2022, the nursing engagement score remained high at 80%.

## Discussion

This small community hospital is the first known to implement an EM program using trained mobility technicians, an established safe patient handling program, and an enhanced BMAT. This model was associated with improvements in patient outcomes, reduced staff injury rates, and consistently high staff engagement levels. The program implementation provides an example of a successful model of EM and supports the feasibility that nurse administrators from smaller institutions can successfully and cost-effectively implement their own EM programs. In 2018, an earlier implementation of an EM program with mobility technicians saved 4.5 hours in a 12-hour shift for nursing staff.^11^ These hours could then be rededicated to other efforts by the nursing staff.

This mobility program should be viewed within the context of the COVID-19 pandemic. The pandemic placed additional pressures on nursing staff, increased staff workload, and substantially increased the volume of patients. As the hospital's patient census nearly doubled, the fall rate increased from 1.849 to 2.459. Once the EM program was implemented, there was a dramatic decrease in the fall rate, likely reflecting the increased attention to EM and safe patient handling. Furthermore, the additional burdens to staff during the COVID-19 pandemic make other findings, including declines in heel and sacral pressure injuries, staff injury, and favorable staff engagement, even more noteworthy.

This program was beneficial to both patients and staff, but it wouldn't have occurred without a CNO to champion its implementation. CNOs are responsible for the delivery of high-quality, cost-effective patient care and play an integral role in promoting any mobility initiative. Challenges to successfully adopting a new EM program include upfront costs, obtaining executive suite buy-in, and establishing staff engagement. Based on these early successes, this hospital has now employed its own internal mobility technician model and will continue to monitor and improve EM initiatives.

## Conclusion

EM can be implemented in any facility provided there's strong support from executive nursing leadership. Adherence to EM programs has historically been a significant issue in hospitals. Deploying a mobility technician model is one strategy to support nursing staff while still encouraging patient mobility. Mobility technicians, when working as part of a team, contribute to improved patient outcomes.

In addition, the use of the technician model with mobility data supported the development of metrics that allowed for a successful and sustainable EM program. The use of relevant metrics and goals are important for replicating the program in other facilities, where they can be customized to meet patient and staff needs. Future research will focus on the impact of this model on additional patient outcomes, such as LOS and delirium.
